# The Prevalence of Mycobacterium Tuberculosis Infection in Saudi Arabia: A Systematic Review and Meta-analysis

**DOI:** 10.1007/s44197-024-00274-w

**Published:** 2024-07-24

**Authors:** Badria Said, Amal H. Mohamed, Ebtihal Eltyeb, Raga Eltayeb, Nagla Abdalghani, Bahja Siddig, Amel Eltahir Banaga Ahmed, Anwar Balla Eltom Ali, Abdulaziz H. Alhazmi

**Affiliations:** 1 Faculty of Medicine, Jazan University, Jazan, 45142 Saudi Arabia; 2 Faculty of Nursing and Health Sciences, Jazan University, Jazan, 45142 Saudi Arabia

**Keywords:** Prevalence, Saudi Arabia, Mycobacterium Tuberculosis, TB

## Abstract

The prevalence of Tuberculosis (TB) serves as a pivotal metric, reflecting the TB burden within a specific demographic. It quantifies the number of individuals affected by either active TB disease or latent TB (LTBI). Such data is crucial for assessing the efficacy of TB control interventions and determining the demand for diagnostic and treatment services. This study aims to consolidate data on TB infection prevalence in Saudi Arabia from existing literature. Additionally, we stratify this prevalence based on age, professional involvement in healthcare, gender, and region. Our search was conducted in PubMed, Scopus, Cochrane Library, and Web of Science databases to determine relevant studies. The pooled prevalence of TB infection among the total population residing in Saudi Arabia was estimated using a random-effect meta-analysis approach and Comprehensive Meta-Analysis software. The protocol was registered in the PROSPERO International Prospective Register of Systematic Reviews, No: CRD42023400984. We included 21 studies, 11 of which were pooled in the analysis. The overall prevalence was 17%.

Regarding the specific population, we found that the prevalence of TB in Saudi Arabia was 9.8% and 26.7% in the general population and the healthcare workers, respectively. Stratifying by age, the highest prevalence was observed in individuals over 50 years (33.0%), while the lowest was in the 10–19 age group (6.4%). In terms of gender, men had a higher prevalence (12.0%) compared to women (9.4%). The prevalence of TB in Riyadh was 6.4%, and 3.6% in Mecca and Medina. Among healthcare workers, nurses and physicians had a prevalence of 14.7% and 15.0%, respectively. Our study found a TB prevalence of 17.0% in Saudi Arabia, higher than the worldwide average of 12.0%. Men had a higher prevalence than women, and healthcare workers had a relatively low prevalence compared to other countries. Age was a significant risk factor, with the highest prevalence in individuals above 50 years. Standardized protocols for screening and diagnosis and targeted interventions are needed to combat TB effectively in the country.

**Supplementary Information:**

The online version contains supplementary material available at 10.1007/s44197-024-00274-w.

## Introduction

Tuberculosis (TB), caused by the *Mycobacterium tuberculosis* bacteria, is a preventable infectious disease that poses a significant health challenge [1, 2]. Before the coronavirus (COVID-19) pandemic, TB prominently held the position of the foremost cause of mortality from a single infectious agent, surpassing even fatalities from Human Immunodeficiency Virus/Acquired Immunodeficiency Syndrome (HIV/AIDS). Without treatment, TB’s mortality rate is nearly 50.0% [3]. A staggering one-quarter of the global population, or roughly 2 billion individuals, is affected by TB. Each year, approximately 10 million people contract the disease, resulting in 1.16 million deaths [4, 5]. Thus, TB remains the most prevalent infectious disease-related cause of death worldwide [1–4]. Approximately one-third of the global population is believed to have a latent TB infection (LTBI). Individuals can carry this dormant form of TB for months or even years without displaying symptoms, but they are at an elevated risk of progressing to active and contagious TB [1]. While humans serve as the sole natural reservoir for *Mycobacterium tuberculosis*, the bacterium’s ability to establish such latent infections has facilitated its widespread transmission [1].

Globally in 2021, there were an estimated 10.6 million acquired TB, and 1.16 million patients died from it (187,000 of them were people living with HIV). Furthermore, there is a 3.6% increase in the incidence rate in 2021 compared to 2020, reversing a nearly 2.0% annual decline over the previous two decades [2, 3, 5]. When an individual is exposed to infection, some TB *mycobacteria* remain in a non-replicating or slowly replicating dormant state for the rest of their life. Many people with this latent form of TB infection may never develop TB disease, and the TB mycobacteria remain inactive for a lifetime without causing the disease. About 5.0 to 10.0% of people with LTBI may develop active TB disease during their lifetime years later when their immune system becomes weak for any reason such as patients with HIV/AIDS or cancers [6]. Bacteriological methods (microscopic smear and culture) are the diagnostic tool for TB, with microscopy with acid-fast bacilli (AFB) staining being the most rapid method. Other testing methods, such as the tuberculin skin test (TST), are sometimes sufficient to accurately identify actual LTBI, especially in high-risk, immuno-suppressed, and BCG-vaccinated individuals [6, 7]. Newer TB blood tests, known as Interferon-Gamma Release Assays (IGRAs), can diagnose LTBI [6–8].

**Fig. 1:**
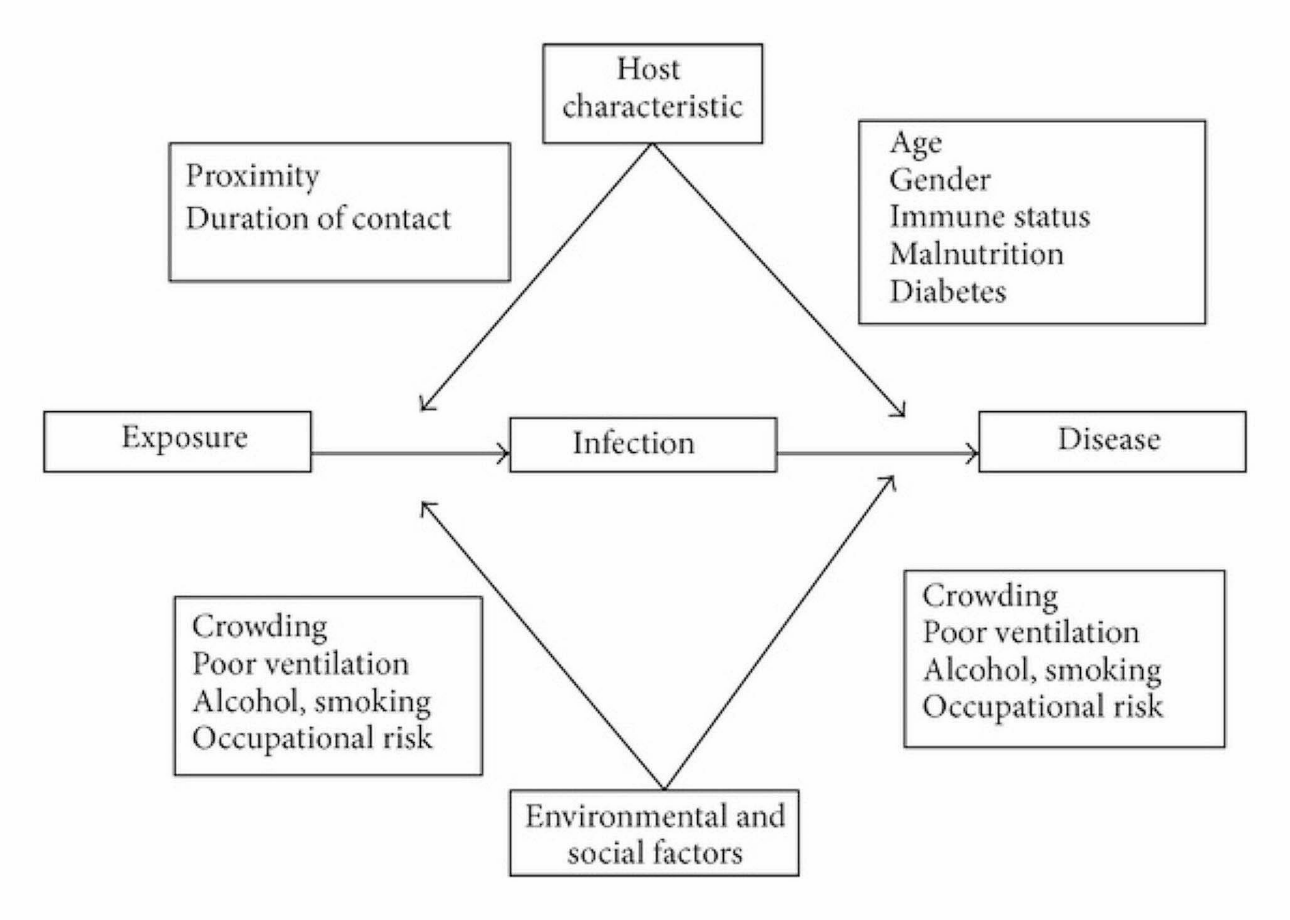
Summary of risk factors of TB

Case rates are usually used by health departments and other organizations to compare the frequency of TB cases in various locations, periods, and demographic classifications. Two categories can be used to separate high-risk groups; people at a greater risk of exposure to the infection or individuals at increased risk of developing TB disease after acquiring the infection [4]. The risk of acquiring an active disease after exposure to tuberculosis bacilli is a two-stage process controlled by external and internal risk factors, including individual, sociodemographic, and geographical predictors, in addition to the bacillary load [9, 10]. A summary of these risk factors is illustrated in the following (Fig. 1) [9].

The most practical methods for lowering the risk of TB complications in high-risk groups, such as close contact, people living with HIV, and healthcare workers, are TB screening and prophylactic therapy. These methods should also be considered in endemic countries to lower the transition from infection to disease. Tools that are extremely sensitive and focused are also necessary for screening for latent TB. Various currently used (and newly developed IGRAs) diagnostic tests for LTBI are highly specific but have decreased sensitivity [9, 11, 12].

Approximately 85.0% of patients can be treated with the currently indicated therapies. Some regimens continue up to months to treat TB infection. To guarantee that everyone who has an illness or infection can receive these therapies, universal health coverage (UHC) is required [2, 3]. According to the 2019 Saudi National Report, the current TB situation in the Kingdom of Saudi Arabia shows that the total number of new and relapsed TB cases was 3004, with an incidence of 8.7 per 100,000 and a therapeutic success rate of (89.9%). The incidence is remarkably higher among non-nationals residing in Saudi Arabia versus national citizens, with 10.9 versus 7.4 per 100,000 population in 2019, respectively. Migrant workers account for 47.0% of smear-positive pulmonary TB cases and 48.0% of extra-pulmonary TB. The non-national population has an incidence rate nearly double that of the Saudi population. Further, males represent approximately 71.2% of the notified cases, any form; and a significant proportion of the patients are young adults (aged less than 45 years), representing approximately 68.6% of notified TB cases, irrespective of gender. Clinically, nearly 86.0% of notified new TB cases in Saudi Arabia are bacteriologically confirmed pulmonary TB [13, 14].

Measuring the burden of TB and early detection of LTBI is critical for planning TB control interventions, assessing their impact on population health, and evaluating whether global targets for reductions in disease burden are achieved. As most TB cases may result from the reactivation of latent infection, identifying people with LTBI who have an increased chance of advancement to active disease is essential to TB control programs. Many studies have been published describing the prevalence of antibiotic resistance of *Mycobacterium tuberculosis* in different parts of Saudi Arabia. Evaluation of these studies is a critical issue in the control of TB. Estimating the epidemiology of different types of TB and drug resistance will establish baseline data which is highly needed for controlling the disease in Saudi Arabia.

## Methods

### Study Design

This systematic review and meta-analysis followed the “Cochrane Handbook” guidelines. The study was reported in strict adherence to the Preferred Reporting Items for Systematic Reviews and Meta-Analyses (PRISMA) guidelines. By adhering to these rigorous standards, we ensured the methodological quality, transparency, and accurate reporting of our study findings [15, 16]. The protocol was registered in the International Prospective Register of Systematic Reviews (PROSPERO) under registration Number: CRD42023400984.

### Literature Search and Eligibility Criteria

Using this search strategy, we searched PubMed, Scopus, Cochrane Library, and Web of Science in July 2023: (“epidemiology” OR “prevalence” OR “incidence”) AND (“tuberculosis” OR “TB” OR “Mycobacterium tuberculosis” OR “M. Tuberculosis”) AND (“Saudi Arabia” OR “Saudi” OR “KSA” OR “Kingdom of Saudi Arabia”). Our criteria for including papers were: (1) Population: population in Saudi Arabia, (2) Outcome: measuring the prevalence of TB in Saudi Arabia, and (3) Study design: Observational, descriptive, and cross-sectional studies. We excluded any studies written in other languages except English or Arabic. Additionally, we excluded reviews, letters, brief reports, and irrelevant studies that did not fulfill the inclusion criteria.

### Study Selection and Data Extraction

Four investigators conducted a comprehensive search, selected relevant studies, and extracted data. Initially, they screened titles and abstracts to ensure alignment with our inclusion criteria. Articles that did not meet these criteria were excluded. Subsequently, the team thoroughly reviewed the full texts of the selected studies.

Regarding data extraction, using a study-specific data extraction form, they collected essential information, including study ID, design, city, nationality, age, gender distribution, diagnostic tests, educational status, the selected population for each study, TB prevalence, and conclusions.

### Operational Terms

Active TB is a disease that is contagious and causes symptoms. The symptoms of active TB mainly depend on whether it is pulmonary or extra-pulmonary. Prevalent symptoms include unexplained weight loss, loss of appetite, fever, chills, fatigue, and night sweats [17]. LTBI is defined as the microbiological identification of TB bacteria with no clinical manifestations. The carrier of TB bacteria who has no symptoms does not feel sick, cannot spread TB bacteria to others, and usually has a positive TB TST or TB blood test [17]. Multidrug-resistant tuberculosis (MDR-TB) is a condition of TB infection caused by bacteria strains that are resistant to therapy with at least two of the first-line anti-TB medications, mainly isoniazid and rifampicin [18].

### Risk of Bias Assessment

To gauge the methodological rigor of the studies, we employed the National Institutes of Health’s (NIH) tool designed for assessing the quality of observational cohort and cross-sectional studies [19]. This tool encompasses a series of questions that probe into the risk of various biases and confounding factors. The reviewers’ assessments were categorized as “good,” “fair”, or “poor” based on the scores obtained from this evaluation. Moreover, we utilized Egger’s test and the funnel plot method to scrutinize the potential presence of publication bias in the included studies.

### Data Synthesis

We pooled TB prevalence in Saudi Arabia as pooled proportions with a 95% confidence interval (CI). The random effect model using the DerSimonian-Laird method was applied. We also investigated TB prevalence among different subgroups, including cities, age groups, healthcare workers, and genders. We investigated the statistical heterogeneity between studies using the I^2^ statistics chi-squared test, with *p* < 0.1 considered heterogeneous and I^2^ ≥ 50% suggestive of high heterogeneity. We conducted statistical analyses using Comprehensive Meta-Analysis Software (CMA).

## Results

**Fig. 2:**
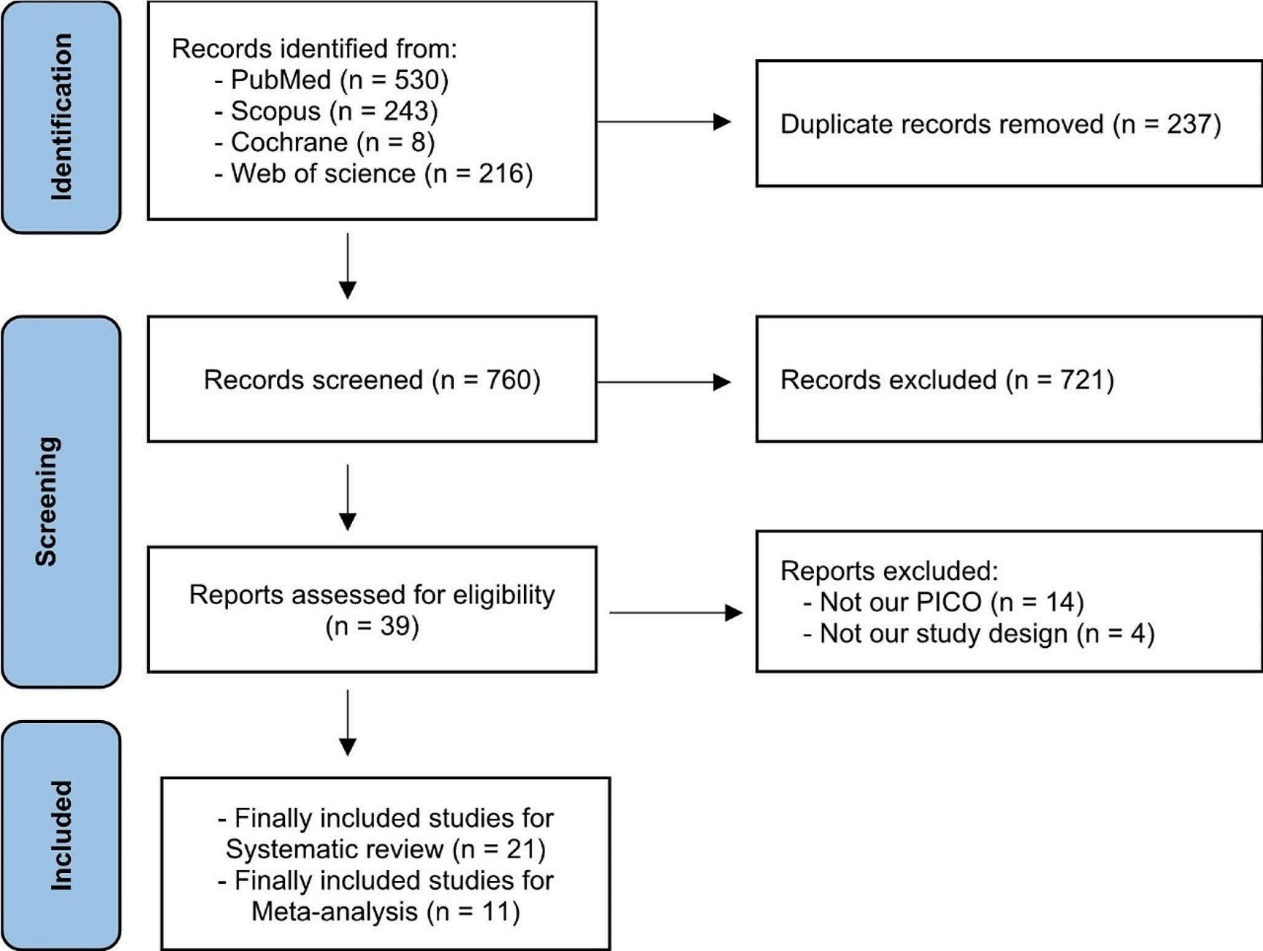
PRISMA 2020 flow diagram for new systematic reviews which included searches of databases and registers only

Nine hundred ninety-seven studies were found after searching four different databases. When we did titles and abstracts screening of the 760 studies after removing duplicates, we found that 721 were unrelated to our study. Then, 39 studies underwent full-text screening, and 21 were finally included; 11 [20–30] were considered for analysis, and 10 studies [31–40] were included as narrative evidence. The PRISMA flow diagram for the selected studies is shown in Fig. 2.

### Study Characteristics and Quality

In our study, 11 studies [20–30] involving 153,245 participants who tested for TB infection were included in the analysis. Most participants were Saudi Arabian, while some patients included in the analysis were of other nationalities. The included studies ranged from 1993 to 2023, providing a wide range of data over several decades. The study designs varied, with most of the studies being cross-sectional, except for two retrospective studies [28, 30]. Various TB tests were utilized across the included papers to diagnose TB. These tests included the Tuberculin Skin Test, TB blood test, IGRA using QuantiFERON TB Gold in the tube (QFT-GIT), and sputum culture (Table 1**)**. Using the NIH tool to assess the quality of the included papers, 12 were determined to have a fair quality rating [20, 21, 24–26, 28, 30, 31, 33–36], while nine studies were deemed poor quality (Supplementary Table 1) [22, 23, 27, 29, 32, 37–40].

### Prevalence of TB in Saudi Arabia

**Fig. 3:**
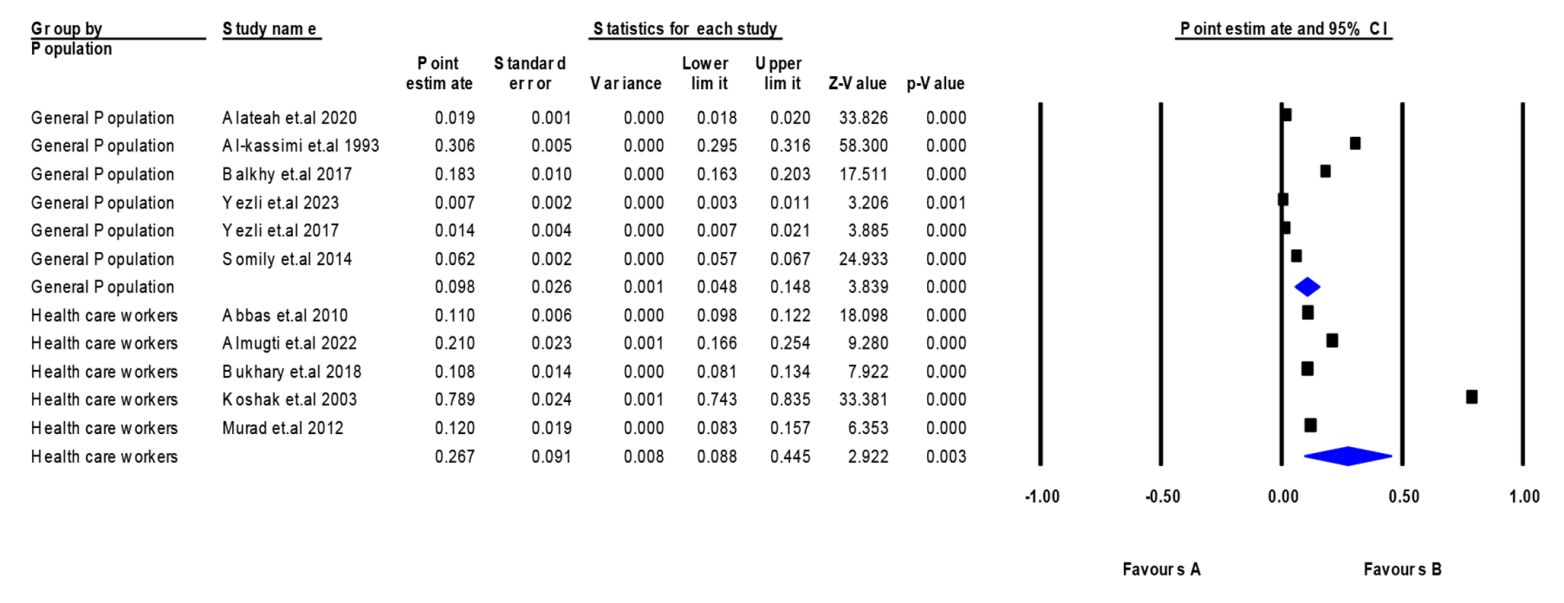
Prevalence of TB in Saudi Arabia

We measured the prevalence of TB in Saudi Arabia by pooling 11 different studies [20–30]. The pooled participants’ prevalence was 17.0% (95% CI: 12.7–21.2%, *p* > 0.00001). Regarding the specific population, we found that the prevalence of TB in Saudi Arabia in the general population was 9.8% (95% CI: 4.8–14.8%), and in the health care workers 26.7% (95% CI: 8.8–44.5%). One of the included papers had a significantly high value that is different from other studies; Koshak et al. 2003 showed a prevalence of 78.9%. The included studies were highly heterogeneous (*p* > 0.00001, I^2^ = 99.99%) (Fig. 3**)**. Most of the studies measured the estimated prevalence of LTBI [20, 22–27], while the remaining four papers [20, 27–29] measured the prevalence of TB and MDR TB. According to Egger’s test with a visual inspection of the funnel plot, we found a risk of publication bias (*p* < 0.00001) (Supplementary Fig. 1).

### Prevalence of TB According to Age

**Fig. 4:**
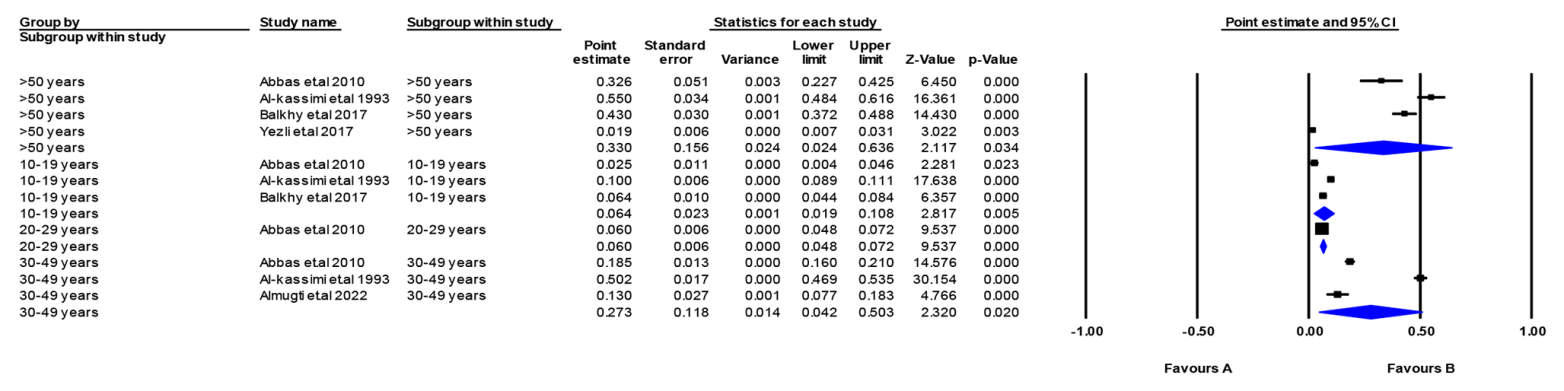
Prevalence of TB in Saudi Arabia (Age Category**)**

We conducted a prevalence assessment of TB in Saudi Arabia, stratifying the analysis by age categories. In the age category of 10 to 19 years, the pooled prevalence was 6.4% (95% CI: 4.4-8.4%, *p* = 0.005), based on data from three studies [20, 22, 24]. The age category of 20–29 years had a pooled prevalence of 6.0% (95% CI: 4.8-7.2%, *p* < 0.00001), which was assessed in a single study [20]. The age category of 30 to 49 years showed a pooled prevalence of 27.3% (95% CI: 4.2-50.3%, *p* = 0.005) based on data from three studies [20, 22, 23]. Lastly, for the age category of more than 50 years, the pooled prevalence was 33.0% (95% CI: 2.4-63.6%, *p* = 0.034), with data from four studies [20, 22, 24, 29]. The overall results were heterogeneous (*p* < 0.00001, I^2^ = 99.17%), indicating significant variability among the studies included in the analysis (Fig. 4).

### Prevalence of TB According to Gender

**Fig. 5:**
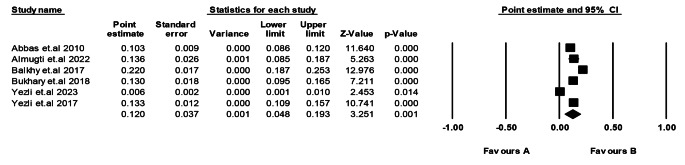
Prevalence of TB in Saudi Arabia among Men

**Fig. 6:**
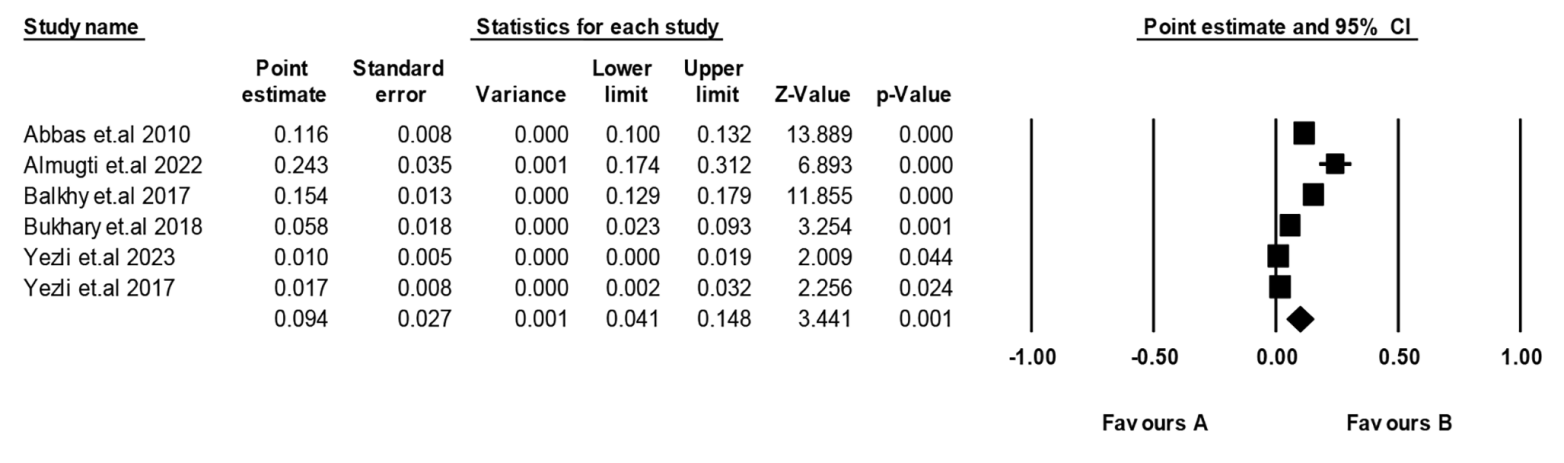
Prevalence of TB in Saudi Arabia among Women

We assessed the prevalence of TB according to gender. Regarding the men subgroup, including six studies [20, 23–25, 29, 30], the pooled participants’ prevalence was 12.0% (95% CI: 4.8–19.3%, *p* = 0.001). The assembled studies for this subgroup were heterogeneous (*p* > 0.00001, I^2^ = 99.99%). We also assessed the prevalence of TB in females by pooling six studies [20, 23–25, 29, 30]. The pooled females’ prevalence was 9.4% (95% CI: 4.1–14.8%, *p* = 0.001). The included studies for this subgroup were also heterogeneous (*p* > 0.00001, I^2^ = 97.9%) (Figs. 5 and 6).

### Prevalence of TB According to the City

**Fig. 7:**
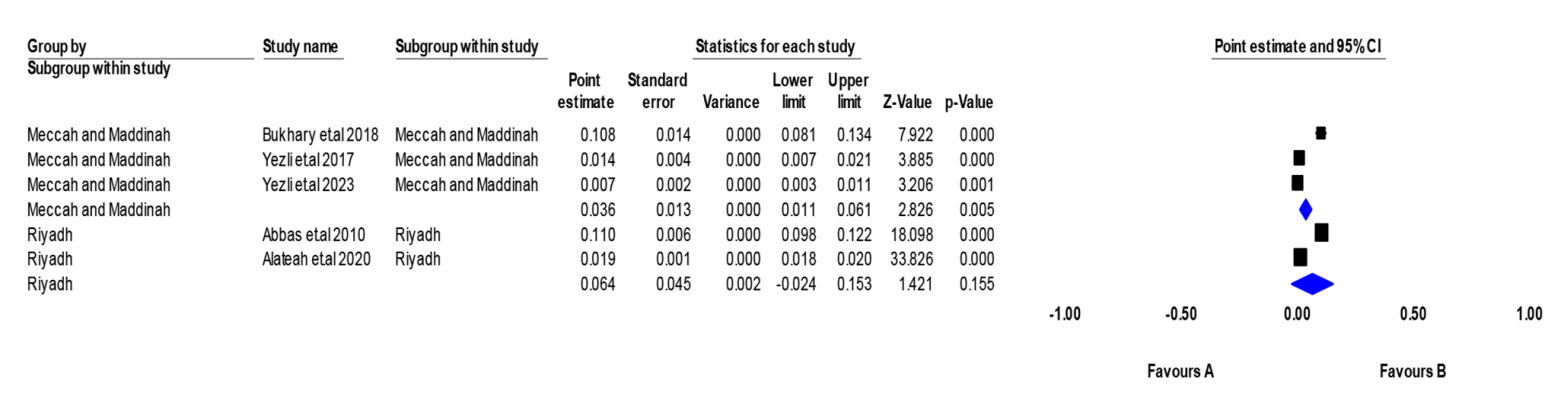
Prevalence of TB in Saudi Arabia in three cities

We assessed the prevalence of TB in the main cities in Saudi Arabia, including Riyadh, Mecca, and Medina, as data were available [20, 21, 25, 29, 30]. The prevalence in Riyadh was assessed by pooling two studies, which was 6.4% (95% CI: 2.4–15.3%, *p* = 0.15). The pooled two studies were heterogeneous (*p* > 0.00001, I^2^ = 99.5%). The prevalence in Mecca and Medina was assessed by pooling three studies, and it was 3.6% (95% CI: 1.1–6.1%, *p* = 0.005). The pooled three studies were heterogeneous (*p* > 0.00001, I^2^ = 96.3%)(Fig. 7**).**

### Prevalence of TB among Healthcare Workers

**Fig. 8:**
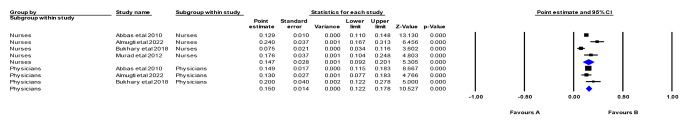
Prevalence of TB in Saudi Arabia among Healthcare workers

We assessed TB prevalence among healthcare workers in Saudi Arabia and split the results into two groups: nurses and physicians, by pooling four studies [20, 23, 25, 27]. The pooled nurses’ prevalence in our study was 14.7% (95% CI: 9.2–20.1%, *p* > 0.00001) [20, 23, 25]. The pooled four studies were heterogeneous (*p* > 0.00001, I^2^ = 82.6%). Our study’s pooled physicians’ prevalence was 15.0% (95% CI: 12.2–17.8%, *p* > 0.00001). The pooled three studies were heterogeneous (*p* = 0.35, I2 = 4.9%) (Fig. 8).

### Qualitative Analysis

**Fig. 9:**
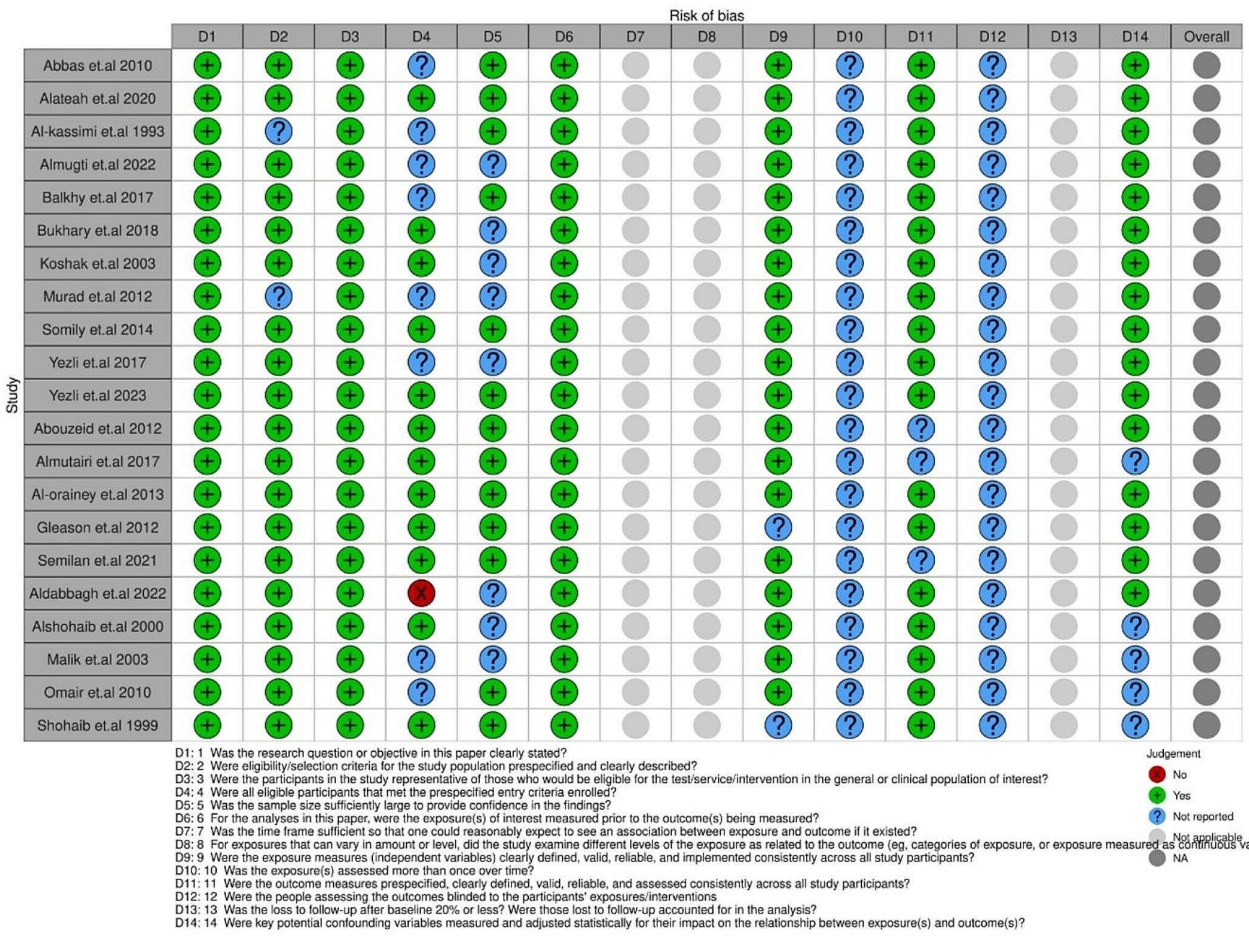
Risk of bias

Abouzeid et al. [31] found that from the year 2000 up to the year 2009, there was an increase in the number of TB-infected cases, especially the non-Saudi residents in Saudi Arabia. The study also found that the incidence in Saudi males is higher than in females, similar to our findings. Almutairi et al. [32] evaluated the incidence rate from 2005 up to 2012 and found that the incidence rate has decreased since the start of 2005 as it was 15.80 per 100,000 populations in 2005 and decreased to 13.16 per 100,000; similar results were found in Al-Orainey et al. [33] as the incidence rate ranged 14–17 per 100,000 decreased to 12.2 per 100,000. Gleason et al. [34] assessed the incidence rate for non-Saudi residents in Makkah, which has a high number of non-Saudi populations, and found that the incidence rate decreased in the non-Saudi population from 2005 to 2009. Two studies [39, 40] had a dialysis population, and the incidence rates were significantly different according to the hospital and preventive measures used. Aldabbagh et al. [36] included patients who received chemotherapy and found that chemotherapy patients have a higher TB prevalence than the general Saudi population. Omair et al. [38] included patients suffering from HIV and their TB incidence of 1354 per 100,000 yearly, which was much higher than in the general population reaching about 30 folds, in addition to the higher mortality rate in HIV patients compared to the average population [41]. The included studies' publication and risk of bias are illustrated in Fig. 9.

**Table 1:** Summary and baseline characteristics of the included studies

Study ID	Study design	City	Geographical Nationality, *n* (%)	Age, *n* (%) y	Males, *n* (%)	Diagnostic test, *n* (%) (If applicable	Educational status, *n* (%)	Assessed population	Prevalence or Incidence of TB	Type of TB	Participants (Total) number	Conclusion
**Abbas et al. 2010**	Cross-sectional study	Riyadh	a. Saudi, 1254 (47.3%) b. Middle East, 285 (10.7%) c. East Asia, 981 (37.0%) d. Western, 91 (3.4%) e. Sub-Saharan, 36 (1.3%)	a. 10-19y, 203 (7.6%) b. 20-29y, 1425 (53.7%) c. 30-39y, 716 (27.0%) d. 40-49y, 220 (8.3%) e. >=50y, 86 (3.2%)	1180 (44.5%)	TST, 2650 (100%)	-	Health care workers	Prevalence = 0.11	LTBI	2650	LTBI was prevalent among recently employed HCWs in Riyadh tertiary hospitals. Definitive programs for detection and treatment of LTBI should be encouraged.”
**Alateah et al. 2020**	Retrospective study	Riyadh	-	Among + ve cases, a. 0-14y, 18 (1.6%) b. 15-29y, 214 (19.1%) c. 30-43y, 175 (15.5%) d. 44-59y, 224 (20.0%) e. >=60y, 492 (44.0%)	672 (60.0%)	TB specimen: smear, culture, and PCR	-	General Population	Prevalence = 0.0193	TB & MDR- TB	58,141	A low prevalence of TB and MDR-TB among study patients over 17 years. A nationwide assessment is needed to understand the TB burden across Saudi Arabia clearly.
**Al-kassimi et al. 1993**	Cross-sectional study	Five provinces in Saudi Arabia	a. Saudi, 5768 (74.6%) b. Non-Saudi, 1953 (25.3%)	a. 5-14y, 2795 (36.2%) b. 15-24y, 1728 (22.4%) c. 25-34y, 1260 (16.3%) d. 35-44y, 919 (11.9%) e. 45-64y, 818 (10.6%) f. 65 + y, 201 (2.6%)	4038 (52.3%)	TST, 7721 (100%) Sputum culture	1. None, 2988 (38.7%) 2. Primary, 3412 (44.2%) 3. Secondary, 950 (12.3%) 4. Higher, 371 (4.8%)	General Population	Prevalence = 0.30566	LTBI	7721	The economic status and availability of free treatment for active cases have led to a comparatively low prevalence rate of tuberculin sensitivity in children. The foci of increased prevalence in the Western Province need special screening arrangements.
**Almugti et al. 2022**	Cross-sectional study	-	1. Saudi, 204 (63.0%) 2. Non-Saudi, 120 (37.0%)	a. 20-30y, 172 (53.0%) b. 31-40y, 144 (44.0%) c. 41-50y, 8 (3.0%)	176 (54.0%)	1. TST, 311 (96.0%) 2. QuantiFERON blood test, 13 (4.0%)	-	Health care workers	Prevalence = 0.21	LTBI 19.0% TB 2.0%	324	There was a low acceptance and completion rate of LTBI therapy among HCWs. Low knowledge about some clinical facts of LTBI, the long duration of treatment, and the fact that the treatment is optional in Saudi health institutes were all barriers to accepting and completing the treatment of LTBI. These factors should be addressed to increase the compliance rate to LTBI treatment.
**Balkhy et al. 2016**	Cross-sectional study	Three provinces	1. Central region, 763 (55.7%) 2. Eastern region, 313 (22.9%) 3. Western region, 293 (21.4%)	a. <15y, 591 (43.2%) b. 15-44y, 502 (36.7%) c. 45-64y, 204 (14.9%) d. ≥65y, 72 (5.3%)	597 (43.6%)	TST and QuantiFERON	1. Illiterate, 175 (13.2%) 2. Primary school, 559 (42.3%) 3. Mid/high school, 455 (34.4%) 4. University and above, 134 (10.1%)	General Population	Prevalence = 0.183	LTBI	1369	The overall agreement of TST and QFTGIT for detecting LTBI among the Saudi general population was 88.8%. QFTGIT is probably comparable to TST for detecting LTBI in an intermediate-burden country with high at-birth BCG vaccination coverage.
**Bukhary et al. 2018**	Cross-sectional study	-	1. Saudi, 221 (42.5%) 2. Non-Saudi, 299 (57.5%)	35.4 (SD 9.9)	348 (66.9%)	TST and QuantiFERON	-	Health care workers	Prevalence = 0.1077	LTBI	520	The consensus between the tests was poor. QFT-GIT detected LTBI when TST was negative in HCWs who had a record of tight contact with TB patients
**Koshak et al. 2003**	Cross-sectional study	Jeddah	1. Saudi, 40 (13.4%) 2. Non-Saudi, 258 (86.6%)	37.8 (SD 7.9)	58 (19.5%)	TST	-	Health care workers	Prevalence = 0.789	LTBI	298	To enhance the protection of healthcare employees and hospitalized patients, adequate preventive actions and annual tuberculin testing of healthcare employees should be regarded.
**Murad et al. 2012**	Cross-sectional study	Jeddah	Saudi, 296 (100%)	-	-	TST	Students, 296 (100%)	Health care workers	Prevalence = 0.12	LTB	296	The prevalence of a positive TST was high among students when considered a primary diagnostic method for latent *Mycobacterium tuberculosis* infection. Strengthening infection control measures is recommended during students’ health care training.
**Somily et al. 2014**	Retrospective study	Riyadh	1. Saudi (80.3%) 2. Non-Saudi, (19.7%)	-	-	Culture	-	General Population	Prevalence = 0.0005	Active TB 6.0% MDR TB 0.7%	9405	Most patients (87%) were Saudis showing an incident rate of 55.6/100,000. The 18–35 age group noticed an increase in TB cases. Resistance to isoniazid was 10.6%, 1% to rifampicin, 2–8% to ethambutol, and streptomycin was 6%.
**Yezli et al. 2017**	Cross-sectional study	Mecca	1. Afghanistan, 316 (27.1%) 2. Bangladesh, 222 (19.1%) 3. Nigeria, 176 (15.1%) 4. Pakistan, 302 (25.9%) 5. South Africa, 148 (12.7%)	a. <=47y, 302 (27.3%) b. >47–55y, 275 (24.9%) c. >55–64y, 296 (26.8%) d. >64y, 233 (21.1%)	823 (72.2%)	Sputum culture Xpert MTB/RIF assay	1. No formal education, 597 (51.7%) 2. Primary education, 163 (14.1%) 3. Secondary education, 217 (18.8%) 4. University-higher education, 178 (15.4%)	General Population	Prevalence = 0.014	Active TB	1063	Further studies are required to define the scale of the TB situation during the Hajj gathering and the need for proactive screening, treatment, and prevention guidelines
**Yezli et al. 2023**	Prospective Cross-sectional study	Mecca	1. South Asia, 734(48.7%) 2. Former Soviet Union, 213 (14.1%) 3. Other African Countries, 355 (23.6%) 4. North Africa, 205 (13.6%)	a. ≤47y, 328 (24.3%) b. >47–55y, 266 (19.7%) c. >55–64y, 384 (28.4%) d. >64y, 373 (27.6%)	1050 (72.0%)	Sputum culture Xpert MTB/RIF assay	1. No formal education, 878 (60.6%) 2. Primary education, 202 (13.9%) 3. Secondary education, 199 (13.7%) 4. University/higher education, 170 (11.7%)	General Population	Prevalence = 0.007	Active TB	1458	International mass gatherings may play an essential part in the international epidemiology of TB. Preventive actions should be mandated to reduce the risk of TB importation and transmission during Hajj and other similar events.
**Abouzeid et al. 2012**	Retrospective study	Najran, Riyadh, Mecca and Tabuk	1. Saudi, 18,859 (50.8%) 2. Non-Saudi, 18,256 (49.2%)	-	-	-	-	General Population	Incidence = 2.7/100,000	Active TB	37,115	TB is a significant public health problem in the KSA, affecting all ages, Saudis and non-Saudis. Recognizing the significant risk factors related to the persistently elevated TB rates in workers migrating to KSA is needed. Additional studies are needed to delineate whether such patients re-activate latent *Mycobacterium tuberculosis* infection or acquire new TB infection after coming to KSA. Proper interventions are needed to decrease TB incidence rates, as have been enforced by other countries.
**Almutairi et al. 2018**	Cross-sectional study	Asir, Baha, Eastern Region, Hail, Jawf, Jazan, Medina, Mecca, Najran, Northern Borders, Qassim, Riyadh, Tabuk	-	-	-	By either positive culture or smear	-	General Population	Incidence = 15.5/100,000	Active TB	32,435	For adequate prevention and control, TB screening should be enforced for non-Saudi workers at ports of entry, and laboratory screening capacity for TB should be evaluated.
**Al-orainey et al. 2013**	Cross-sectional study	Central, Western, Eastern, Northern, Southern, Mecca and Jazan	1. Saudi, 33,460 (52%) 2. Non-Saudi, 30,885 (48%)	-	-	-	-	General Population	Incidence = 15.3/100,000	Pulmonary TB 73.0% Extrapulmonary TB 27.0%.	64,345	TB control is facing some challenges in several regions of the Kingdom. NTP must evaluate and improve TB control strategies to reduce disease incidence to elimination levels.
**Gleason et al. 2012**	Retrospective study	All 13 cities	1. Saudi, 10,783 (52.5%) 2. Non-Saudi, 9769 (47.5%)	-	-	-	-	General Population	Incidence = 1. Saudi, 12.18/100,000 2. Non-Saudi, 27.66/ 100,000	TB	20,552	Disparate KSA regional and longitudinal TB trends existed from 2005 through 2009 by Nationality. A review of all TB policies in KSA that addresses screening for LTBI, drug resistance, and a new TB public health education program is recommended.
**Semilan et al. 2021**	Cross-sectional study	Makkah, Jeddah, Taif, AlQunfotha	1. Saudi, 416 (36.6%) 2. Non-Saudi, 720 (63.4%)	35.47 (SD 12.96)	838 (68.2%)	-	-	General Population	Incidence = 9/100,000	TB	1270	The most increased incidence rate of TB among works supporting basic engineering could be described by tight contact with the general population in closed spaces for long periods, and low socioeconomic status
**Aldabbagh et al. 2022**	Cross-sectional study	Jeddah	Saudis, 203 (100%)	57.32 (SD 13.85)	78 (38.0%)	Interferon-gamma release assays	-	Cancer Patients Receiving Chemotherapy	Prevalence = 0.2	LTBI 68.0% Active TB 32.0%	203	TB prevalence was more elevated in chemotherapy patients than general Saudi population. Patients with solid tumors and older had more risk of acquiring the infection, signifying the importance of preventing TB and malignancy coexistence by initiating screening policies in cancer patients.
**Alshohaib et al. 2000**	Prospective cohort study	Jeddah	1. Saudi, 6 (75.0%) 2. Non-Saudi, 2 (25.0%)	Cases: 45 (SD 8.485)	3 (37.5%)	TST, chest radiograph, and sputum examinations	-	Patients with chronic renal failure	Prevalence = 0.1	Active TB	80	Regular screening for TB is suggested for patients with chronic renal failure at Renal Units and TB chemoprophylaxis should be regarded for those undergoing hemodialysis, particularly in countries with a high incidence of TB.
**Malik et al. 2003**	Retrospective study	Riyadh, Al-Dammam, Al-Noor, Gizan, Samta, Tabuk, Ar Ar, AlJouf	-	38 to 58y	75 (56.8%)	Radiology, Smear, and Cultures	-	Dialysis Patients	Prevalence = 0.073	Active TB	1791	TB was not the immediate cause of death in any of the patients except one, in whom it could be contributory.
**Omair et al. 2010**	Retrospective cohort study	-	Saudis, 217 (100%)	29.7 (SD 14.7)	132 (60.8%)	TST	-	People living with the human immunodeficiency virus (HIV)	Incidence = 1354/100,000	TB in HIV patients	217	Among PLWH in Saudi Arabia, TB incidence is 30 times higher than in the general population, with significant mortality despite early diagnosis, treatment, and tertiary care support.
**Shohaib et al. 1999**	Retrospective study	Jeddah	Among cases: 1. Saudi, 9 (52.9%) 2. Non-Saudi, 8 (47.1%)	30 to 75	-	Smear Culture	-	Dialysis Patients	Prevalence = 0.081	Active TB	210	For diabetic patients presenting for dialysis, in areas with high endemicity for TB, chemoprophylaxis with anti-TB agents should be considered.

SD = Standard deviation; TB = Tuberculum bacilli; TST = Tuberculin skin test; QFT-GIT = QuantiFERON TB Gold in tube; Y = year; N = Number; HCW = Health Care Workers; LTBI = Latent TB infection. MTB = Mycobacterium tuberculosis; KSA = Kingdom of Saudi Arabia; ARTI= Acute respiratory tract infection

## Discussion

TB is widespread throughout the world, and it remains a significant public health and human development concern, particularly in developing countries. Despite the efficacy of directly observed therapy (DOT) in decreasing TB transmission and its progression, poverty reduction and socioeconomic development play a significant role in reducing and preventing TB infection [42]. The Sustainable Development Goals (SDGs) and the World Health Organization’s (WHO) End TB Strategy have optimistic aims for putting an end to TB infection [2, 42]. We found that the total prevalence of TB in Saudi Arabia is17.0%. Saudi Arabia is considered an important country with visitors and tourists for religious purposes due to the presence of the two holy sites in Mecca and Medina regions. Thus, the mass gathering (during Umrah and the annual Hajj) is linked to a higher risk of infectious diseases, particularly respiratory tract infections. Also, the increased prevalence of TB can be influenced by migration. About 60.0% of all new TB cases occur in the top six TB-endemic nations: India, Indonesia, China, Nigeria, Pakistan, and South Africa. Massive migration forces may have an impact on the dynamics of TB transmission. The 2016 worldwide TB report shows a broad burden, with Kuwait dominating the list among Gulf Health Council (GHC) countries with 200 cases per million and the UAE coming in last with 6.8 cases per million [43].

Regarding factors associated with TB epidemiology in Saudi Arabia, we found that the prevalence of TB in men is higher than the prevalence in women in Saudi Arabia as the results were 12.0% and 9.4%, respectively, which is known as the incidence of infection with TB in males is almost double the incidence in females as it was confirmed by WHO annual reports [2]. Further, we measured the prevalence of TB in physicians and nurses, and we found that their prevalence is close to the general population as it was 15.0% and 14.7%, and both were almost equal. The percentage of healthcare workers’ infections with TB in Saudi Arabia is lower when compared to other countries in Asia, such as Indonesia, which has a prevalence of 25.0% from a recent study [44], in addition to another meta-analysis that yielded results that the prevalence in health care workers was 28.0% [45]. The relatively lower TB prevalence among healthcare providers in Saudi Arabia may be attributed to the cautionary attitudes and strict adherence to preventive measures, as demonstrated by previous studies highlighting the significance of these measures in reducing the incidence of TB [46, 47]. One study by Koshak et al. [26] revealed an unusually high prevalence rate (using a positive TST) compared to other existing research, as they reported a prevalence of 78.9%, significantly higher than that of other studies. Specifically, they found a prevalence of 60.0% among Saudi Arabian citizens and 81.8% among non-Saudi nationalities. It is worth noting that this high prevalence can be explained by the specific population under investigation, which comprises healthcare workers at King Abdul-Aziz University Hospital. This unique group might explain the elevated percentage in comparison to other studies.

TB in the main cities, including the capital and holy cities, was not high, as the prevalence was 3.6% in Mecca and Medina, while the prevalence was 6.4% in Riyadh. Regarding different age categories, the highest prevalence was observed among individuals above 50 years, with a prevalence rate of 33.0%. The second-highest prevalence was found in the age group ranging from 30 to 49 years, with a prevalence rate of 27.3%. In contrast, the individuals aged 10 to 19 exhibited a significantly lower prevalence rate of 6.4%. This lower prevalence in the younger age group may be attributed to lesser contact with TB-infected individuals and possibly the persistence of the effective protection that is gained from TB vaccination which is obligatory in Saudi Arabia. Furthermore, the compromised immune system in older individuals may make them more vulnerable to TB infection and its subsequent prevalence [48]. These findings highlight the importance of age as a TB risk factor and the need for targeted interventions and preventive measures, especially among the old age population. Efforts to enhance TB control and prevention should consider age-specific approaches to address the higher prevalence observed in older age groups.

Regarding clinical practice, this study can provide important insights into the prevalence of TB infection in Saudi Arabia. Additionally, it can shed light on the incidence of TB among healthcare providers and determine if they face a higher infection risk than the general population. Understanding the specific risk factors healthcare providers face can help implement targeted preventive measures. Moreover, the study can offer valuable information on whether or not regions such as Mecca and Medina, which host Hajj and Umrah pilgrimages, have a higher TB prevalence than other parts of the country. This knowledge can assist in developing appropriate strategies to manage and control TB transmission during these significant religious gatherings. Furthermore, the study can identify the age category with the highest TB prevalence. This information can guide public health interventions by enabling the implementation of age-specific preventive measures and targeted screening efforts. By focusing on the age group most affected by TB, the healthcare system can optimize resource allocation and enhance TB control efforts in Saudi Arabia.

Our study possesses several notable strengths. Firstly, we are the first meta-analysis to determine the prevalence of TB in Saudi Arabia. This groundbreaking aspect of our study contributes novel insights to the existing literature. Moreover, by conducting subgroup analyses, we provided a more comprehensive assessment of TB prevalence across different comparison groups, enhancing our findings’ robustness. Lastly, our study covers a significant time frame, allowing for a comprehensive overview of TB trends in the country over an extended period. This temporal perspective provides valuable insights into the long-term patterns of TB prevalence in Saudi Arabia.

However, this study bears several limitations. Firstly, we could not include the prevalence of TB in vulnerable groups like infants, children under the age of ten years, and pregnant ladies due to finite resources in terms of available studies. Secondly, the small population size in the analysis relative to the extended time frame may affect the consistency of our findings. Additionally, the quality of the included studies varied from poor to fair, with no high-quality studies identified, which introduces the potential for bias in our analysis. Furthermore, the heterogeneity of the included studies, particularly in the diagnostic test used to determine the existence of TB, could not be solved due to the limited available data. These limitations indicate the need for future studies with larger sample sizes, higher-quality designs, and similar diagnostic methods for TB. In addition, future research should prioritize assessing socioeconomic factors in patients with TB, as this information can significantly impact disease management and outcomes.Furthermore, our study examined the epidemiology in some regions of Saudi Arabia, which may not reflect the overall epidemiological data in the country. Our study represents mainly the prevalence among physicians and nurses, whereas few data were found for other healthcare providers, which adds another limitation to our study.

## Conclusion

In conclusion, our study revealed essential insights into the epidemiology of TB infection in Saudi Arabia country. While the overall prevalence of TB in our study was17.0%, higher than the worldwide prevalence of 12.1%, it remained relatively low compared to other Asian countries. The higher prevalence in men compared to women is consistent with global trends and is supported by the World Health Organization’s annual reports. Notably, the prevalence of TB among healthcare workers in Saudi Arabia was found to be relatively low compared to other countries. This might be attributed to the adherence to preventive measures. Our findings also highlighted age as a significant risk factor for TB, with the highest prevalence observed among individuals above 50. This underscores the importance of age-specific interventions and preventive measures, especially for the elderly.

## Electronic Supplementary Material

Below is the link to the electronic supplementary material.

Supplementary Material 1

Supplementary Material 2

Supplementary Material 3

## Data Availability

The data are available on request from the corresponding author.
