# Supplementary material for: The Prevalence of Mycobacterium Tuberculosis Infection in Saudi Arabia: A Systematic Review and Meta-analysis
Source: J Epidemiol Glob Health. 2024 Jul 24;14(3):658–76. doi: 10.1007/s44197-024-00274-w (PMC11442870; doi:10.1007/s44197-024-00274-w)
Supplement: Supplementary file 1 — Supplementary Material 1 [file 44197_2024_274_MOESM1_ESM.docx]

| **ID** | **NIH Quality Assessment Tool for Observational Cohort and Cross-Sectional Studies** | | | | | | | | | | | | | | | **Quality rating:  good (11-14) or  fair  (7.5-10.5)  or (0-7),  Yes = 1 // No = 0.5 // NR & NA & CD = 0** |
| --- | --- | --- | --- | --- | --- | --- | --- | --- | --- | --- | --- | --- | --- | --- | --- | --- |
|  | **1. Was the research question or objective in this paper clearly stated?** | **2. Were eligibility/selection criteria for the study population prespecified and clearly described?** | **3. Were the participants in the study representative of those who would be eligible for the test/service/intervention in the general or clinical population of interest?** | **4. Were all eligible participants that met the prespecified entry criteria enrolled?** | **5. Was the sample size sufficiently large to provide confidence in the findings?** | **6. For the analyses in this paper, were the exposure(s) of interest measured prior to the outcome(s) being measured?** | **7. Was the time frame sufficient so that one could reasonably expect to see an association between exposure and outcome if it existed?** | **8. For exposures that can vary in amount or level, did the study examine different levels of the exposure as related to the outcome (eg, categories of exposure, or exposure measured as continuous variable)?** | **9. Were the exposure measures (independent variables) clearly defined, valid, reliable, and implemented consistently across all study participants?** | **10. Was the exposure(s) assessed more than once over time?** | **11. Were the outcome measures prespecified, clearly defined, valid, reliable, and assessed consistently across all study participants?** | **12. Were the people assessing the outcomes blinded to the participants' exposures/interventions?** | **13. Was the loss to follow-up after baseline 20% or less? Were those lost to follow-up accounted for in the analysis?** | **14. Were key potential confounding variables measured and adjusted statistically for their impact on the relationship between exposure(s) and outcome(s)?** | **total scores** |  |
|  | **Yes / No / Not reported (NR) or cannot determine (CD) or not applicable (NA)** | **Yes / No / Not reported (NR) or cannot determine (CD) or not applicable (NA)** | **Yes / No / Not reported (NR) or cannot determine (CD) or not applicable (NA)** | **Yes / No / Not reported (NR) or cannot determine (CD) or not applicable (NA)** | **Yes / No / Not reported (NR) or cannot determine (CD) or not applicable (NA)** | **Yes / No / Not reported (NR) or cannot determine (CD) or not applicable (NA)** | **Yes / No / Not reported (NR) or cannot determine (CD) or not applicable (NA)** | **Yes / No / Not reported (NR) or cannot determine (CD) or not applicable (NA)** | **Yes / No / Not reported (NR) or cannot determine (CD) or not applicable (NA)** | **Yes / No / Not reported (NR) or cannot determine (CD) or not applicable (NA)** | **Yes / No / Not reported (NR) or cannot determine (CD) or not applicable (NA)** | **Yes / No / Not reported (NR) or cannot determine (CD) or not applicable (NA)** | **Yes / No / Not reported (NR) or cannot determine (CD) or not applicable (NA)** | **Yes / No / Not reported (NR) or cannot determine (CD) or not applicable (NA)** |  |  |
| **Abbas et.al 2010** | Yes | Yes | Yes | NR | Yes | Yes | NA | NA | Yes | NR | Yes | NR | NA | Yes | 8 | Fair |
| **Alateah et.al 2020** | Yes | Yes | Yes | Yes | Yes | Yes | NA | NA | Yes | NR | Yes | NR | NA | Yes | 9 | Fair |
| **Al-kassimi et.al 1993** | Yes | NR | Yes | NR | Yes | Yes | NA | NA | Yes | NR | Yes | NR | NA | Yes | 7 | Poor |
| **Almugti et.al 2022** | Yes | Yes | Yes | NR | NR | Yes | NA | NA | Yes | NR | Yes | NR | NA | Yes | 7 | Poor |
| **Balkhy et.al 2017** | Yes | Yes | Yes | NR | Yes | Yes | NA | NA | Yes | NR | Yes | NR | NA | Yes | 8 | Fair |
| **Bukhary et.al 2018** | Yes | Yes | Yes | Yes | NR | Yes | NA | NA | Yes | NR | Yes | NR | NA | Yes | 8 | Fair |
| **Koshak et.al 2003** | Yes | Yes | Yes | Yes | NR | Yes | NA | NA | Yes | NR | Yes | NR | NA | Yes | 8 | Fair |
| **Murad et.al 2012** | Yes | NR | Yes | NR | NR | Yes | NA | NA | Yes | NR | Yes | NR | NA | Yes | 6 | Poor |
| **Somily et.al 2014** | Yes | Yes | Yes | Yes | Yes | Yes | NA | NA | Yes | NR | Yes | NR | NA | Yes | 9 | Fair |
| **Yezli et.al 2017** | Yes | Yes | Yes | NR | NR | Yes | NA | NA | Yes | NR | Yes | NR | NA | Yes | 7 | Poor |
| **Yezli et.al 2023** | Yes | Yes | Yes | Yes | Yes | Yes | NA | NA | Yes | NR | Yes | NR | NA | Yes | 9 | Fair |
| **Abouzeid et.al 2012** | Yes | Yes | Yes | Yes | Yes | Yes | NA | NA | Yes | NR | NR | NR | NA | Yes | 8 | Fair |
| **Almutairi et.al 2017** | Yes | Yes | Yes | Yes | Yes | Yes | NA | NA | Yes | NR | NR | NR | NA | NR | 7 | Poor |
| **Al-orainey et.al 2013** | Yes | Yes | Yes | Yes | Yes | Yes | NA | NA | Yes | NR | Yes | NR | NA | Yes | 9 | Fair |
| **Gleason et.al 2012** | Yes | Yes | Yes | Yes | Yes | Yes | NA | NA | NR | NR | Yes | NR | NA | Yes | 8 | Fair |
| **Semilan et.al 2021** | Yes | Yes | Yes | Yes | Yes | Yes | NA | NA | Yes | NR | NR | NR | NA | Yes | 8 | Fair |
| **Aldabbagh et.al 2022** | Yes | Yes | Yes | No | NR | Yes | NA | NA | Yes | NR | Yes | NR | NA | Yes | 7.5 | Fair |
| **Alshohaib et.al 2000** | Yes | Yes | Yes | Yes | NR | Yes | NA | NA | Yes | NR | Yes | NR | NA | NR | 7 | Poor |
| **Malik et.al 2003** | Yes | Yes | Yes | NR | NR | Yes | NA | NA | Yes | NR | Yes | NR | NA | NR | 6 | Poor |
| **Omair et.al 2010** | Yes | Yes | Yes | NR | Yes | Yes | NA | NA | Yes | NR | Yes | NR | NA | NR | 7 | Poor |
| **Shohaib et.al 1999** | Yes | Yes | Yes | Yes | Yes | Yes | NA | NA | NR | NR | Yes | NR | NA | NR | 7 | Poor |
