# Supplementary figures and images for: The Prevalence of Mycobacterium Tuberculosis Infection in Saudi Arabia: A Systematic Review and Meta-analysis
Source: J Epidemiol Glob Health. 2024 Jul 24;14(3):658–76. doi: 10.1007/s44197-024-00274-w (PMC11442870; doi:10.1007/s44197-024-00274-w)

**Funnel Plot of Standard Error by Point estimate**

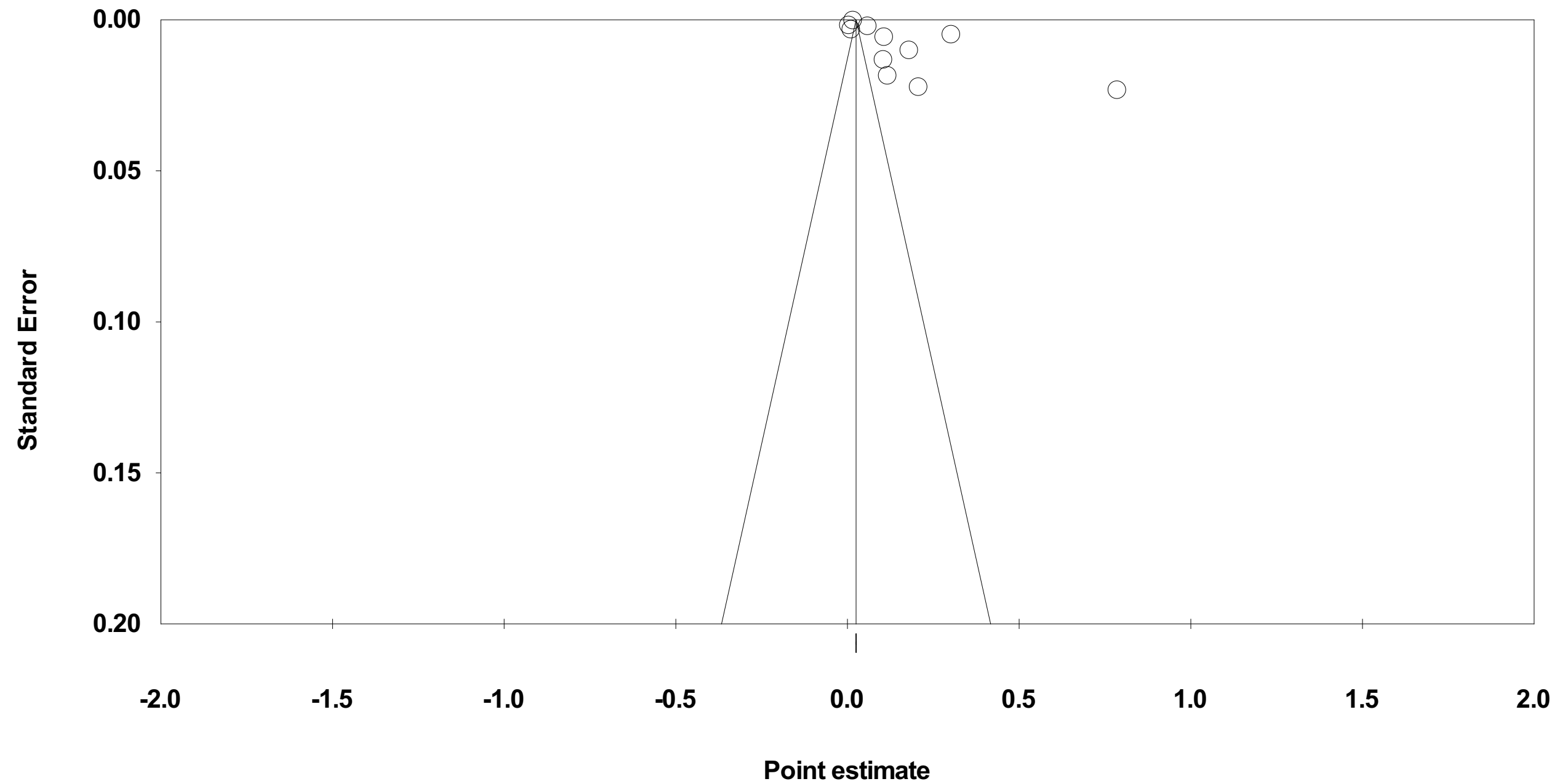

Supplement: Supplementary file 3 — Supplementary Material 3 [file 44197_2024_274_MOESM3_ESM.pdf]
